# A latent transition analysis of bullying and victimization in Chinese primary school students

**DOI:** 10.1371/journal.pone.0182802

**Published:** 2017-08-24

**Authors:** Yiqin Pan, Hongyun Liu, Puiyi Lau, Fang Luo

**Affiliations:** 1 Department of Psychology, Beijing Normal University, Beijing, China; 2 Beijing Advanced Innovation Center for Future Education, Beijing, China; 3 Institute of Criminology, University of Cambridge, Cambridge, United Kingdom; Radboud Universiteit, NETHERLANDS

## Abstract

Bullying is a social phenomenon that impacts a large number of children and young people, worldwide. This study aimed to longitudinally examine the development of bullying and victimization in Chinese students in grades 4, 5, and 6. We used latent class analysis to empirically identify groups of youth with different bullying and victimization patterns, and then used latent transition analysis to explore the movement of children between these latent classes over time. Results showed that: (1) across the three time points, students could be classified into four classes: bullies, victims, bully-victims, and non-involved children; and (2) students in the non-involved class tended to remain in that class when moving to higher grades, students in the bully and victims classes tended to transition to the non-involved class, while students in the bully-victims class tended to transition to the bullies class. Thus, future intervention should be implemented to prevent bully-victims from bullying behaviors.

## Introduction

Bullying is defined as repeated acts of aggression through which an individual (or group of individuals) intentionally harms someone physically, verbally, or psychologically. Furthermore, it occurs regularly over time, is intentional, and involves an imbalance in power between the bully and the victim [1–3]. Bullying is common in primary and secondary schools worldwide, with estimated rates ranging from 15–25% among various countries, including Australia, Austria, England, Finland, Germany, Norway, and the United States [4]. Due to a lack of attention to the phenomenon, the problem of school bullying in China was also serious in recent years. The prevalence rate of bullying at schools in Mainland China ranged from 2% to 34%; while victimization was more prevalent, ranging from 2% to 66% [5]. The media reported more than 20 vicious school bullying events in 2016. For example, a girl in Zhejiang province slapped another girl 40 times in 20 seconds and a boy in Hubei province hit the head of another boy 24 times with a steel pipe. The seriousness of these acts led the Chinese government to draw up “The Guidance on the Prevention of Bullying and Violence in Primary and Secondary Schools” [6] in 2016, in order to control school bullying.

Both bullying and victimization are associated with a host of adjustment difficulties in childhood and adolescence. While victimized students may suffer from a range of psychosomatic problems (e.g., high levels of anxiety) [5, 7], academic difficulties (e.g., reduced academic performance) [8] and health problems (e.g., headaches) [9], research has shown that bullies also face a number of adjustment difficulties (e.g., substance abuse and excessive drinking) [5, 10]. Thus, the unique group of students who both bully others and are bullied by others (bully-victims) may face compounded difficulties. Indeed, research has shown anxiety, depression, and psychosomatic symptoms to be equally common among bullies and victims, and most common among bully-victims [11]. Bullying is clearly a phenomenon that negatively impacts children and warrants research attention.

Most studies of bullying were conducted with Western populations and relatively few studies have focused on bullying in China. However, the findings from Western populations may not be generalized to bullying and victimization in China. Previous research has shown that children in China (as a representative country of Eastern culture) show greater objection to bullying and more sympathy to victims than do children in Britain (as a representative country of Western culture) [12]. Moreover, Bergeron and Schneider found that, relative to individualistic cultures (e.g., the United States), collectivist cultures (e.g., China) show lower levels of bullying [13]. It seems to follow that Chinese students would suffer from less bullying than would students in Western countries.

Bullying problem develops with age [14]. Research showed that in Western countries, bullying behavior decreased before students entered middle school (grades 4 and 5), but increased after students entered middle school (grade 6); while victimization decreased over time [15]. However, the studies conducted with Chinese populations were mostly cross-sectional in nature. For example, Shao, Liang, Yuan, and Bian classified the first-year junior high students in Hangzhou to four groups: aggressive children (16.2%), victimized children (27.4%), aggressive victimized children (9.2%), and non-involved children (47.2%) [16]. Nonetheless, this study did not demonstrate how students moved from one class to another over time and, to the authors’ knowledge, no previous research investigated the development of bullying in the Chinese educational system. Longitudinal research on the development and transition of Chinese students between bullying and victimization classes is warranted.

One typical approach that researchers have used to categorize students into subgroups is using one or two standard deviations (SD) above or below the mean of bullying and victimization as a cut-off for the groups of bullies, victims, bully-victims, and non-involved children [17]. While this approach can effectively relate categories to degrees, there is no standard procedure for determining the number of groups, the location of cut-off points, or the difference implied between a student whose score is a little higher than the cut-off point and one whose score is a little lower than the cut-off point, making class meaning unclear. A second and more recently developed approach, which overcomes the abovementioned disadvantage, is latent class analysis (LCA). LCA is a multivariate statistical model that explores the underlying (rather than the observed) grouping variable through the use of categorical and continuous indicators [16, 18] (LCA which only involves continuous indicators is also called latent profile analysis [19]). LCA can be longitudinally extend to latent transition analysis (LTA) which defines the movement of individuals among latent classes across different time points. For example, Nylund examined the peer victimization experiences of approximately 1,300 urban public school students in grades 6, 7, and 8. Results showed that when students moved between victim groups, they were most likely to move from a more victimized group to a less victimized group [20].

LTA can be applied to student bullying and victimization behaviors to determine the way in which students transition from one class to another. The method uses a measurement model to create groups and a structural model to describe change in group membership over time, and this allows researchers to explore the development of groups by examining the probabilities of transition. Finally, LTA uses a flexible model specification, enabling a detailed description of the development of bullying and victimization. The method can verify whether transition probabilities are the same across transition points, and it can also detect a higher-order effect. A first-order effect refers to the effect of the status at time point *i* on the status at time point *i*+1; for example, the effect of class membership in grade 4 on class membership in grade 5. A higher-order effect refers to the effect of the status at time point *i* on the status at time point *i*+*n* (*n*>1); for example, the lasting and direct effect of class membership in grade 4 on class membership in grade 6.

The present study explored the development of bullying and victimization classes in China. Specifically, we first measured the bullying and victimization experiences of primary school students in grades 4, 5, and 6. We then applied LCA to each time point in order to categorize students into classes. Finally, we applied LTA to study the transition between bullying and victimization classes throughout the primary school years. This study had two main goals: first, to identify the latent classes characterized by both bullying and victimization using LCA on peer nominations data; and second, to examine how children moved between these classes over time. More specifically, the research goals included: (1) classifying students on the basis of their bullying and victimization experiences and interpreting the characteristic of each class; (2) exploring the long-term transition of students between the subgroups over 3 years; (3) examining the stability of the transition between the three time points; and (4) examining the higher-order effects of the classes in the first time point on the last time point. We made four hypotheses. First, that there would be four groups across all grades: bullies, victims, bully-victims, and non-involved children. Second, on the basis of Zhang’s finding that the ratio of victims to bullies decreased in line with children’s age in primary school [3], we hypothesized that school bullying and victimization would be alleviated over time. Third, as the students would not undergo a school transition during this time period, we hypothesized that they would be likely to have a similar school environment across the two transition points; thus, we hypothesized that the rate of students’ transitions between classes would be the same across the transition points. And fourth, as the transition between classes over the three time points was a developmental process, we hypothesized that the classes in grade 4 would have a higher-order effect on the classes in grade 6.

## Method

### Participants

The participants were primary school children from 22 classes in 6 schools across Beijing. We performed a longitudinal study, following the same children from grade 4 through grades 5 and 6, conducting one investigation each year. Some participants were unavailable for all three investigations, which resulted in a sample of 712 participants (375 boys and 337 girls) in grade 4, with an average age of 10.95; 669 participants (351 boys and 318 girls) in grade 5, with an average age of 11.96; and 685 participants (356 boys and 329 girls) in grade 6, with an average age of 12.94.

The study procedure was approved by the Ethics Committee at Beijing Normal University and we obtained written informed consent from the guardians of the students enrolled in our study. Data for this study (presented in S1 File) came from a project supported by the National Funds of Social Science, aiming at analyzing the development of children’s misconduct without intervention from parents and teachers, using advanced longitudinal methods. The study began in 2012. Students in grade 4 were invited to take part in the study in June 2012, with the consent of headmasters, parents, and children. These children were given questionnaires to report on bullying and victimization behaviors. Twelve months later (when they were in grade 5), they were asked to fill in the same questionnaire. Another 12 months later (when they were in grade 6), they were again asked to fill in the same questionnaire.

### Measures

#### School bullying and victimization scale

This eight-item scale (adapted from Chang’s study [21]) measures the types and frequencies of children’s bullying and victimization in schools and is administered using peer nomination. Bullying is assessed using four items, including “classmates who quarrel with other classmates,” “classmates who push other classmates,” “classmates who speak ill of other classmates,” and “classmates who infuriate other classmates.” Victimization is also assessed using four items, including “classmates who are always pushed,” “classmates who are always teased,” “classmates who are always spoken ill of,” and “classmates who are the object of gossip.” When the items are presented in Chinese and used in the Chinese context, the terms match the Chinese definition of bullying and victimization. Previous studies have shown the measure to have satisfactory reliability when used with Chinese children [21, 22]. For the bullying items, Cronbach’s alpha ranges from .918 to .929 across the three time points; for the victimization items, Cronbach’s alpha ranges from .852 to .903. For the peer nomination procedure, students are asked to nominate up to three classmates who best correspond to the item description. The number of nominations of each student against each item is then counted. This method is especially suitable for measuring peer interaction between students. In addition, compared to self-reporting, peer nominations better avoid the social desirability effect, leading to more accurate measurement.

#### Age

In the present study, each student was asked to report his or her age.

### Statistical analysis

Given that the present research applied a measure that drew on peer nomination, class size impacted upon the numeric results (i.e., a student who was unanimously considered aggressive by his or her entire class could be nominated 25 or 125 times, depending on the class size). Thus, the number of nominations was converted into a percentage of the class, and these percentages were used in subsequent analyses.

#### Latent class analysis

An exploratory cross-sectional LCA was performed for grade 4, grade 5, and grade 6 separately in the following steps: First, we determined the optimal number of latent classes, with one to five latent class models fitted to the data and compared. Second, we used LCA to classify students into classes and interpreted the characteristic of each class.

A combination of statistical indicators was used to determine the best fitting model. We compared models on the basis of four indicators: AIC, BIC, ABIC, and entropy. Lower values of AIC, BIC, and ABIC indicate a better fitting model, and the best fitted model occurs when these indicators reach their lowest value. Entropy indicates a greater certainty of a classification, with higher entropy values indicating a greater accuracy of classification. Further, it was important for the model to provide information in line with the substantive theory of the outcome. Therefore, when comparing measurement models, we considered not only the statistical measures of fit but also the practical implications of the model.

#### Latent transition analysis

Longitudinal LTA was then fitted to the three time points. In the present study, we explored the development of student patterns and their characteristics using the following models:

Model 1: A baseline model was estimated with no parameter restrictions. In this model, all parameters were estimated freely.

Model 2: Assuming that full measurement invariance would facilitate straightforward discussion about transitions between classes, we explored whether full measurement or partial invariance could be assumed across the three time points. In Model 2, we fixed the equality of the parameters of the measurement model. If no difference in model fit was found between Model 1 and Model 2, measurement invariance was assumed.

Model 3: To test whether stationary transition probabilities were applicable, we estimated Model 3. In Model 3, transition probabilities were fixed as equal across transition points (i.e., the transition metric of different patterns between grade 4 and grade 5 was fixed to equal that between grade 5 and grade 6). If no difference was found between the model fits of Model 2 and Model 3, then stationary transition probabilities were applied.

Model 4: To test whether the students’ statuses in grade 4 showed a higher-order effect from the first time point to the last time point, we estimated Model 4 to test for the existence of a second-order effect. In Model 4, when estimating the transition probabilities between grade 5 and grade 6, we considered the statuses of students in grade 4. If no difference was found between the model fits of Model 2 and Model 4, then we determined there to be no second-order effect.

We analyzed the results using Mplus 7.0 [23]. Mplus uses the robust maximum likelihood (RML) estimator to estimate LTA models; it is suitable for non-normal and non-independent data, as it provides robust standard errors.

## Results

### Latent class analysis

Table 1 compares the resulting model fit indices, beginning with the most parsimonious one-class model and proceeding to the five-class model.

**Table 1:** Model fit indices derived from LCA with 1–5 classes.

		1 CLASS	2 CLASSES	3 CLASSES	4 CLASSES	5 CLASSES
Grade 4	AIC	44192.89	41580.90	40824.19	40147.21	39730.09
	BIC	44265.98	41695.10	40979.50	40343.63	39967.63
	ABIC	44215.18	41615.72	40871.54	40207.10	39802.51
	Entropy		0.98	0.961	0.986	0.959
Grade 5	AIC	41837.88	39458.97	38427.42	37658.27	37209.05
	BIC	41909.98	39571.61	38580.62	37852.02	37443.35
	ABIC	41859.17	39492.24	38472.67	37715.49	37278.25
	Entropy		0.981	0.976	0.981	0.977
Grade 6	AIC	43540.26	40970.17	39919.08	39180.17	38679.31
	BIC	43612.73	41083.40	40073.08	39374.94	38914.84
	ABIC	43561.93	41004.02	39965.12	39238.41	38749.74
	Entropy		0.978	0.981	0.98	0.976

Table 1 shows that when increasing the number of classes in grade 4, the AIC, BIC, and ABIC values decreased. However, compared to the increase from three to four classes, the increase from four to five classes did not show a significant decrease in BIC value. Therefore, we balanced the goodness of fit and the number of classes and considered the four-class model optimal. Additionally, the four-class model showed the highest entropy value. This is in line with previous research, which has shown that there are typically four classes of students in school bullying and victimization (e.g., Shao et al., 2014). Therefore, for Grade 4, we selected the four-class model.

The results of the measurement models considered for Grade 5 and Grade 6 are also presented in Table 1. The process used to identify the appropriate number of latent classes for Grade 4 was described in detail above; therefore, the discussion of the process for Grade 5 and Grade 6 is here abbreviated. The model fit results for Grade 5 and Grade 6 were similar to those for Grade 4. Thus, the same decisions were made for Grade 5 and Grade 6. The four-class LCA model was deemed most reasonable for Grade 5 and Grade 6.

**Fig 1:**
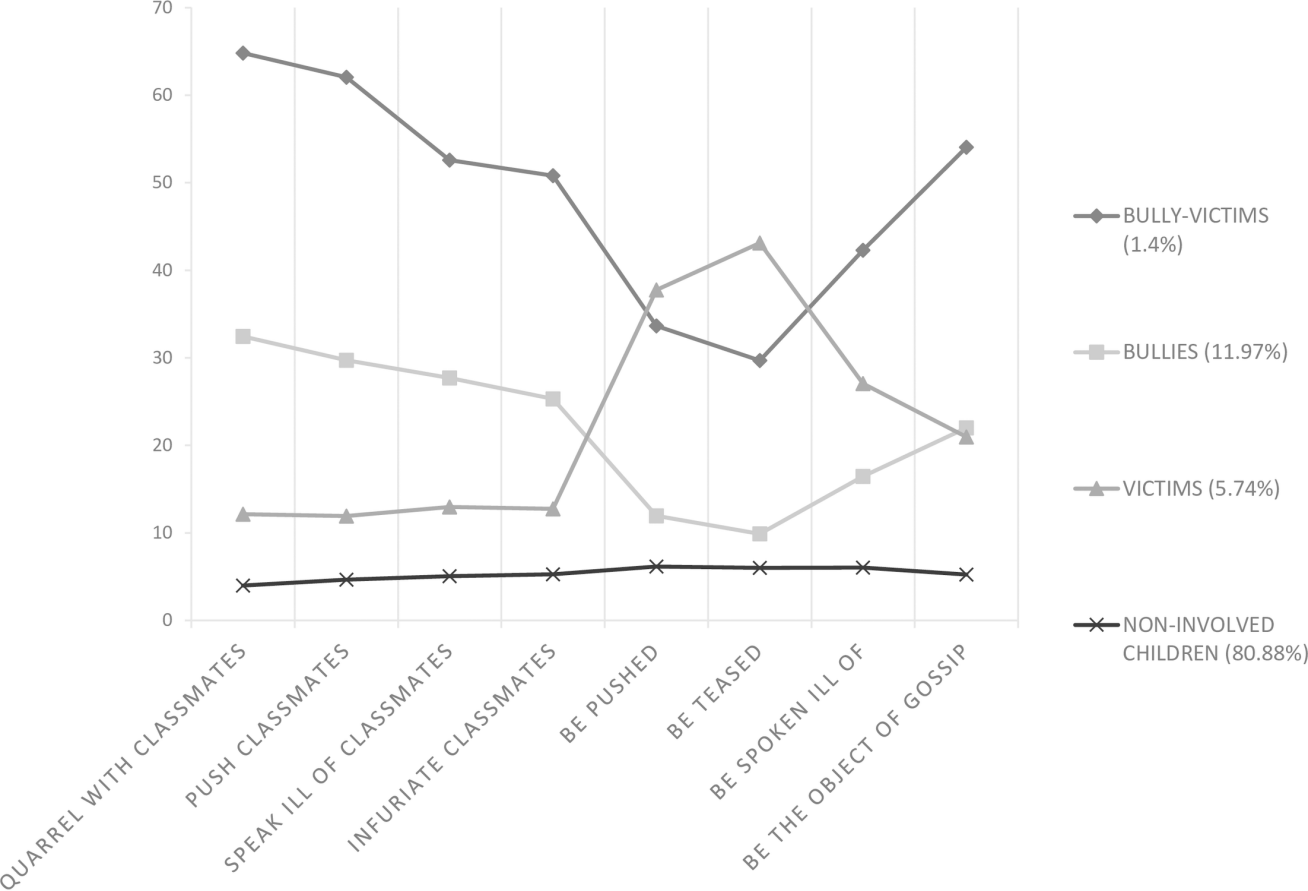
The profile plot for the four-class model for Grade 4 (n = 712).

**Fig 2:**
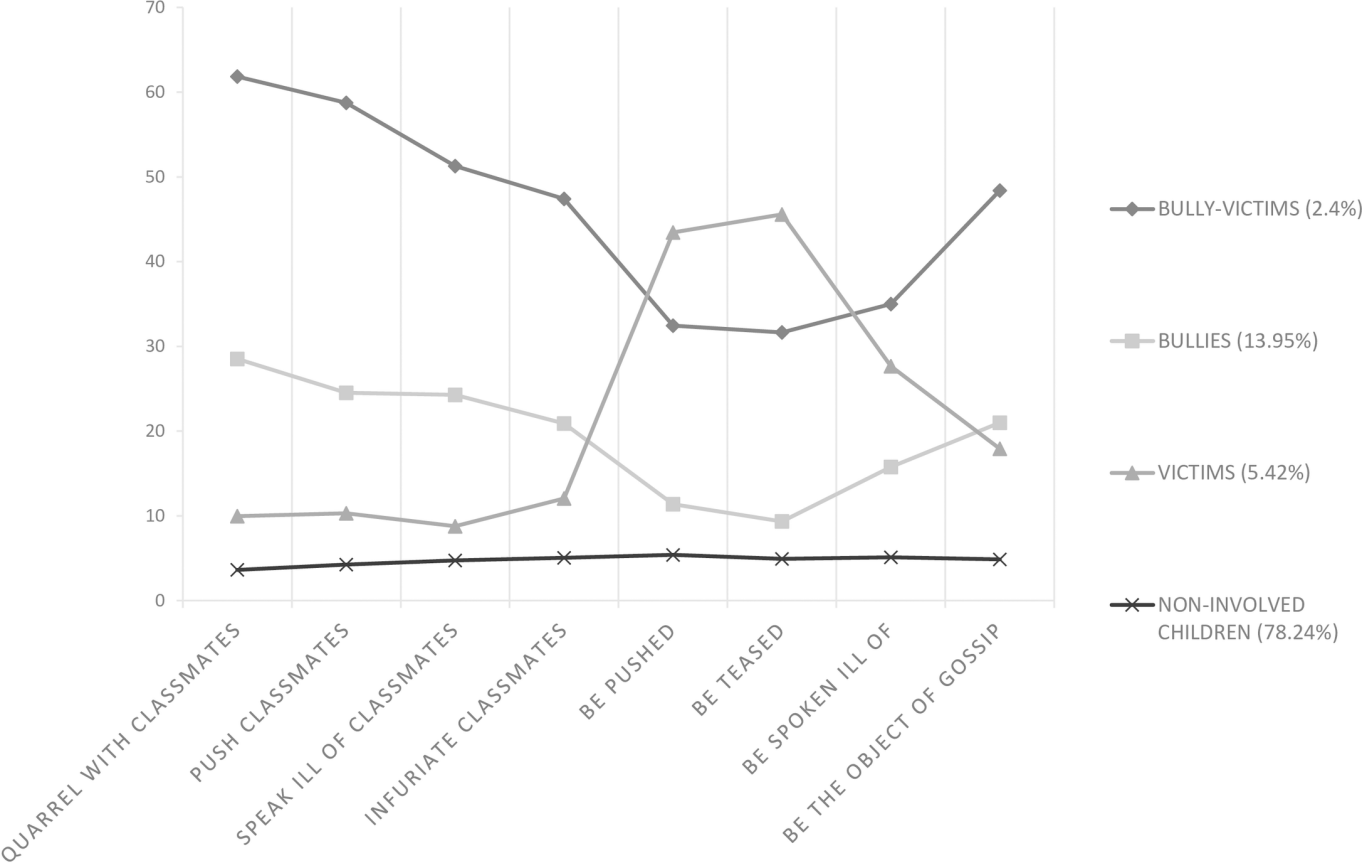
The profile plot for the four-class model for Grade 5 (n = 669).

**Fig 3:**
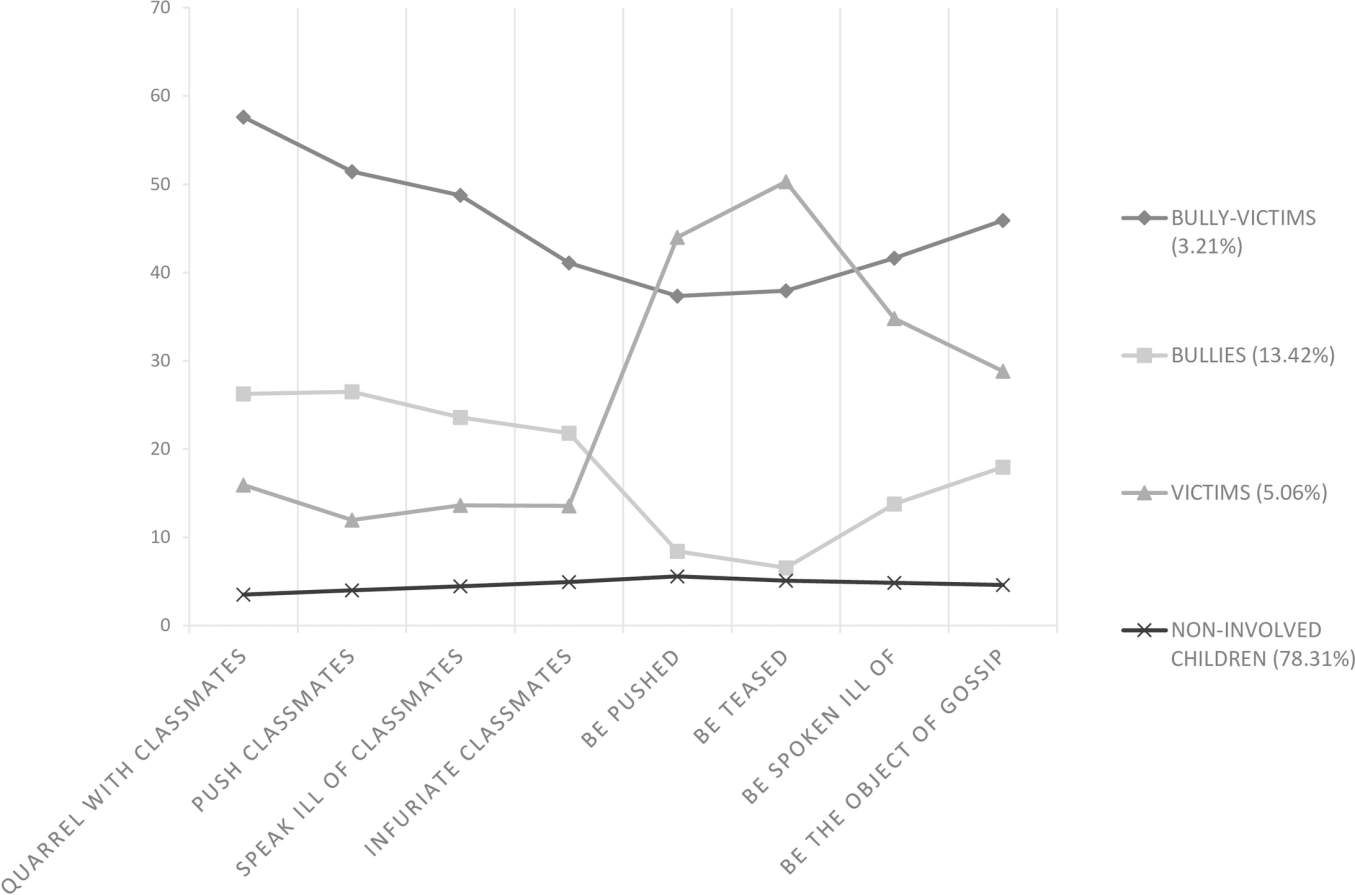
The profile plot for the four-class model for Grade 6 (n = 685).

For LCA models with continuous latent class indicators, the means and variances of the latent class indicators attach substantive meaning to the latent classes. Figs 1, 2 and 3 displays the means of the latent class indicator plots for the four-class solution for Grade 4, Grade 5, and Grade 6. The nine bullying and victimization items are presented along the x-axes of each plot. The y-axes present the means of each item.

The four lines, called profiles, correspond to the four classes in the LCA solution, and the values are the means for each of the eight items across the four classes. Looking at the grade 4 plot which represents 1.4% of the sample, indicates that the individuals in this class had high means for all of the bullying and victimization items. Thus, this class was the “bully-victims” class. The second line, which represents 11.97% of the sample, indicates high means for the bullying items and low means for the victimization items. This describes the “bullies” class. The third line, which represents 5.74% of the sample, indicates low means for the bullying items and high means for the victimization items. This illustrates the “victims” class. The bottom line, which represents 80.88% of the sample, indicates low means for all of the items, and reflects the “non-involved” class.

When looking across grade 4, grade 5, and grade 6, similar profiles can be observed. For both grade 5 and grade 6, we can conclude from Figs 1, 2 and 3 that the students could be divided into the following four class categories: “bully-victims,” “bullies,” “victims,” and “non-involved.” The “bully-victims” class accounted for the smallest percentage of the students and the “non-involved” class accounted for the largest percentage.

### Latent transition analysis

After the measurement model was modeled, the next step used LTA to explore the transitions between latent classes.

The model fit of Model 1 to Model 4 is presented in Table 2. To explore measurement invariance, which refers to the level of difference between the structure of the four classes across time, we compared Model 1 and Model 2, using two models showing complete measurement non-invariance and complete measurement invariance, respectively. The likelihood ratio test (LRT) indicated a significant difference in fit between models (*χ*^*2*^ (64) = 194.466, *p*< .05). However, we proceeded to select Model 2, assuming full measurement invariance for the following reasons: First, the item profile plots were remarkably similar across groups and time, and this consistency provided powerful support for the assumption of full invariance. Second, depending on the nature of the observed outcomes, the length of time considered, and the nature of the construct, measurement invariance may not be realistic, and therefore both theoretical and practical reasons must determine whether measurement invariance should be assumed. And third, in most applications of LTA, full measurement invariance is assumed for practical reasons, because it ensures that the number and structure of classes are the same across time and allows for a straightforward interpretation of transition probabilities. Furthermore, Nylund also assumed full measurement invariance despite finding significant difference between the freely estimated model and the fully constrained model [24].

**Table 2:** The model fits of model 1 to model 4.

	Log likelihood Value	df
M1	-57887.921	147
M2	-57985.154	83
M3	-57987.710	71
M4	-57974.861	92

To study whether the stationary transitions were reasonable, we compared Model 2 and Model 3, using one model that estimated a transition matrix for each transition point (e.g., one for grade 4 to grade 5 and another for grade 5 to grade 6) and another that constrained the transition matrices to be equal across the two transition points. The LRT indicated no significant worsening in fit when equality was imposed (*χ*^*2*^ (12) = 5.112, *p*> .05). In other words, no significant differences in transition probabilities across the two transition points were found.

To determine the existence of a higher-order effect, we compared Model 2 and Model 4. The LRT indicated a significant difference in fit between the models (*χ*^2^ (9) = 20.586, *p*< .05). Therefore, we concluded that there was a significant impact of a higher-order effect.

Table 3 presents the transition probabilities of the model with stationary transition matrices. Transition probability refers to the probability of transition to a certain class at time point *i*+1, given students’ class membership at time point *i*. Values that describe the probabilities of status stability are presented along the diagonal axis, and the off-diagonal values describe the probabilities of movement between classes. Several important patterns emerge from the transition matrices. First, all students were more likely to remain in the same latent class, but especially those in the non-involved class. Second, there was a markedly higher probability that a student would transition to the non-involved class from the bullies and victims classes. Third, compared to the bullies class, the victims class had a higher probability of transitioning to the bully-victims class. Fourth, the bully-victims class had a higher probability of transitioning to the bullies class than to the victims class.

**Table 3:** Transition probabilities for the stationary LTA models.

		Grade 5 (Grade 6*)			
		Bully-victims	Bullies	Victims	Non-involved children
Grade 4 (Grade 5*)	Bully-victims	0.804	0.196	0	0
	Bullies	0.046	0.763	0	0.191
	Victims	0.069	0.005	0.827	0.098
	Non-involved children	0	0.029	0.017	0.954

* The transition probabilities between grades 4 and 5 were the same as those between grades 5 and 6.

## Discussion

The current study had two main goals: first, to identify latent classes characterized by both bullying and victimization, using LCA; and second, to describe the class stability and movement between classes, over time. Results showed four distinctive classes among grade 4 students: bully-victims, bullies, victims, and non-involved children. These groups followed a pattern in which the majority of students were categorized as non-involved, followed by a small group of bullies and victims, and a smaller group of bully-victims. At grade 5, students demonstrated similar categorizations. They were likely to have remained in the same class, though there was some probability for the bullies and the victims to have transitioned to the non-involved class. At grade 6, the same four classes were again obtained: the majority of the students were grouped into the non-involved class, and most students had also undergone a transition that was similar to the transition shown between grades 4 and 5. The grade 4 classes showed a higher-order effect on grade 6, and the classification at grade 6 was largely determined by the status at grade 4.

### School bullying and victimization were stable over time

We found stability across grades 4, 5, and 6 in the classification results, as well as in the transition probabilities across the three time points. First, independent LCA findings showed that the bully/victim profiles were similar over time. The students were classified into four distinctive classes across grades 4 to 6: one class with high item scores on bullying and victimization, one class with high bullying and low victimization scores, one class with low bullying and high victimization scores, and one class with low bullying and low victimization scores. This finding is consistent with classification results from studies of Western populations [15] and with theories based on Western cultures [25].

Second, the LTA results showed no significant difference in the transition probabilities across the three time points. In other words, the transition probabilities between grades 4 and 5 were similar to those between grades 5 and 6. This finding is consistent with that obtained in the United States by Nylund [24], who used LTA analysis to show that students were just as likely to move between the three victim classes (victimized, sometimes victimized, and non-victimized) across two different transition points (between grades 6 and 7 and between grades 7 and 8).

Third, the majority of students appeared to be largely stable within their classes over time. As illustrated by Table 3, the probabilities of status stability were over .75 across all classes, indicating that most of students tended to remain in that class when moving to higher grades. This result is inconsistent with that obtained by Nylund [24], which indicates that movers rather than stayer consisted the majority of students. The inconsistency might result from culture differences between two countries, as China students devoted themselves largely to prepare for College Entrance Examination during adolescence while US students tended to place importance to personality development in this period.

This finding illustrates the stable nature of bullying and victimization in Chinese students. Thus, societal, familial and educational interventions are required to tackle problems of bullying in China.

### The proportion of bullying and victimization classes decreased

Overall, examination of the transition probabilities showed that school bullying tended to decrease over time. This is because most students in the non-involved class tended to remain in the same class and a small number of students–particularly those in the bullies or victims classes–tended to move to the non-involved class.

First, the LTA results showed that most students in the non-involved class were likely to remain in the non-involved class. This finding is consistent with that of Nylund [24], who conducted LTA on victimized groups across grades 6, 7, and 8 and classified students into the following three classes: victimized, sometimes victimized, and non-victimized. The transition probabilities based on these three classes showed that students tended to remain in the same group at adjacent time points.

Second, students in both bullies and victims classes exhibited a relatively high probability of transitioning into the non-involved class. This finding is similar to that of Zhang: in a sample of 9,205 primary and junior school children in urban and rural China, Zhang found that the ratio of victims to bullies decreased with children’s age, and, relative to the decrease in the ratio of victims to bullies from grade 4 to grade 5, the decrease from grade 5 to grade 6 was less significant [3]. This result is also similar to that of studies conducted in Western populations: Williford et al. found that bullying decreased between grades 4 and 5, before the students entered middle school [15]; Nylund found that victimization decreased between grades 6 and 8 [24]. One explanation for the decrease in bullying is that victims may develop social interaction strategies (such as submissive behavior) to cope with the conflicts posed by bullies over time, and therefore no longer become bullied [26]. As for bullies, Olweus argued that, as children mature, they develop stronger social skills and may be better able to get along with their peers and empathize with victims [27]. Together, these theories may explain the decrease in bullying behavior over time.

### A noteworthy class: The bully-victims class

The present study used LCA to explore the classes in school bullying and victimization problem and identified one of the latent classes as the bully-victims class in all three grades. This is similar to Shao et al.’s findings of four groups of adolescents in China (grade 7 students): “general” children, “aggressive” children, “victimized” children, and “aggressive victimized” children [16]. “Aggressive victimized” children have also been considered a unique group of victims in studies of Western populations, with similar groups being labeled “provocative victims” [28] and “aggressive victims” [29, 30].

Interestingly, our results show that students in the bully-victims class tended to transition to the bullies class over time. The probability that those in the bully-victims class would remain in this class was 0.804, and the probability that they would transition to the bullies class was 0.196. However, the possibility that they would transition to the victims or the non-involved classes was 0. This finding indicates that some bully-victims may change from students who both bully others and are bullied by others to students who only bully others, and is consistent with previous studies of Western populations. In a sample of 3,932 adolescents, Barker et al. conducted trajectory analysis from early- to mid-adolescence and found that about 3% of the students maintained a high degree of bullying and experienced relatively severe victimization, while 2% maintained a high degree of bullying but showed decreased levels of victimization [31].

This finding highlights that researchers should consider bullying and victimization simultaneously, in order to gain a more comprehensive picture, to better distinguish between heterogeneous groups of students, and to offer more precise classifications. Previous studies, which have investigated bullying and victimization separately, are limited, as their bullies and victims groups have included bully-victims, who have different characteristics from bullies and victims. Therefore, the findings of these studies are confounded and the possible distinct etiology of bully-victims remains unexplored. For instance, the causes of bullying-victimization differ to some degree from the causes of bullying and victimization. It has been suggested that the parents of bullies may sometimes be hostile. By contrast, parents of victimized children may be overprotective. However, bully-victims come from homes in which parents are less involved with their children and are sometimes rejecting [4], weak family attachment serves as a reliable predictor for bully-victims [32]. Therefore, bully-victims apparently face different psychological problems from bullies and victims. Comprising a special class involving both bullying and victimization, these students require more attention from researchers.

### Limitations

The present research suffers from some limitations that must be improved upon. First, the number of items was small: we used only eight items to measure bullying and victimization, and thus we could not capture all types of peer bullying and victimization. The measurement method of peer nomination restricted the number of items. Although peer nomination is an optimal method for measuring peer interaction between students, compared to a Likert scale, it requires participants to devote more time and energy to consider the status of all group members. If the number of items is too large, the measurement effect is affected. In the future, researchers could apply more items to conduct a broader measurement of bullying and victimization, by collecting data several times.

Second, due to limited funding, our data were collected from only 6 schools in Beijing and further research is required to generalize the findings to other parts of China. Additionally, nearly 80% of our sample comprised one large class (the non-involved children class), whereas the bully-victim class probably only consisted of about 25 participants (3% of 700). Having small classes in LTA may limit meaningful interpretation of our data, particularly with regards to transition probabilities. Thus, further research to investigate school bullying using a more representative sample size is warranted.

## Conclusion

Using latent transition analysis, the study found that bullying and victimization in Chinese primary schools had a tendency to decrease from grade 4 to grade 6. However, relative stability of class membership was also found, which called for educational, familial and societal interventions to tackle the bullying phenomenon. Bully-victims represented a noteworthy group, as students in this group were more likely to transition to the bullies class, rather than the non-involved class, over time. Thus, intervention should be implemented to prevent bully-victims from transferring to bullying behaviors.

## Supporting information

S1 File Data underlying the findings. (SAV)
